# Prevalence of Vitamin D Deficiency Among Children With Tuberculosis and Its Relationship With Demographic and Clinical Characteristics

**DOI:** 10.7759/cureus.110559

**Published:** 2026-06-09

**Authors:** Debajyoti Banerjee, Braham P Kalra, Kiran Bhat, Varuna Jethani, Dimple Bamrah

**Affiliations:** 1 Department of Pediatrics, Himalayan Institute of Medical Sciences, Dehradun, IND; 2 Department of Pediatrics, Subharti Institute of Medical Sciences, Dehradun, IND; 3 Department of Biochemistry, Himalayan Institute of Medical Sciences, Dehradun, IND; 4 Department of Respiratory Medicine, Himalayan Institute of Medical Sciences, Dehradun, IND; 5 Department of Obstetrics and Gynecology, Himalayan Institute of Medical Sciences, Dehradun, IND

**Keywords:** 25-hydroxy vitamin d, children, india, pediatric tuberculosis, tuberculosis, uttarakhand, vitamin d deficiency

## Abstract

Background

Tuberculosis (TB) continues to be a significant contributor to childhood morbidity and mortality in high-burden countries, including India. Host nutritional status plays an important role in susceptibility to *Mycobacterium tuberculosis*, particularly through the modulation of cell-mediated immunity. Beyond its classical role in calcium homeostasis, vitamin D has important immunomodulatory effects, including enhancement of macrophage activity, promotion of phagosome-lysosome fusion, and induction of antimicrobial peptides such as cathelicidin. Although several studies in adults have reported an association between vitamin D deficiency and active TB, pediatric data, particularly from the Indian subcontinent, remain limited. The present study was conducted to determine the prevalence of vitamin D deficiency among children with TB and to examine its relationship with selected demographic and clinical characteristics.

Methods

This prospective study was conducted over a 12-month period and included 41 children aged ≤18 years who were newly diagnosed with TB. Sociodemographic and clinical data were collected using a structured proforma. Serum 25-hydroxyvitamin D (25(OH)D) concentrations were measured using a chemiluminescence assay and expressed in nmol/L. Vitamin D status was categorized as deficient (<50 nmol/L) or non-deficient (≥50 nmol/L). The distribution of vitamin D levels was analyzed according to age group, sex, socioeconomic status, TB diagnosis category, monthly family income, and diagnostic method.

Results

Among the 41 children with TB, vitamin D deficiency was observed in 37 (90.2%) participants, while only 4 (9.8%) had non-deficient vitamin D levels. The mean serum vitamin D level was 34.75 ± 11.28 nmol/L. Pulmonary TB was the most common diagnosis (41.5%), followed by abdominal TB (29.3%), central nervous system (CNS) TB (19.5%), and disseminated TB (9.7%). Vitamin D deficiency was observed across all age groups and socioeconomic strata. However, no statistically significant association was found between vitamin D deficiency and age group (p = 0.524), sex (p = 0.762), socioeconomic status (p = 0.908), TB diagnosis category (p = 0.640), monthly family income (p = 0.790), or diagnostic method (p = 0.730). Pearson correlation analysis showed weak, non-significant correlations between vitamin D levels and age (r = 0.082, p = 0.610), as well as monthly family income (r = 0.088, p = 0.584).

Conclusions

This study demonstrated a high prevalence of vitamin D deficiency among children diagnosed with TB. While no statistically significant association was identified between vitamin D deficiency and the demographic or clinical parameters assessed, the findings highlight the frequent occurrence of inadequate vitamin D levels in pediatric TB. Larger prospective studies are needed to further clarify the role of vitamin D in childhood TB and to explore the potential therapeutic value of vitamin D supplementation.

## Introduction

Tuberculosis (TB), an infectious disease caused by *Mycobacterium tuberculosis*, continues to be a major contributor to global morbidity and mortality. Despite ongoing international control programs, TB remains a significant public health concern, especially in high-burden countries such as India, which has the highest number of TB cases globally, accounting for approximately 27% [1-3]. In endemic regions, childhood TB contributes approximately 10%-20% of total TB cases and serves as an indicator of ongoing community transmission [4]. Globally, TB was responsible for over 239,000 childhood deaths in 2015, highlighting the substantial vulnerability of the pediatric population. The diagnosis of TB in children is particularly challenging due to its paucibacillary nature, nonspecific clinical presentation, and difficulty in obtaining adequate microbiological samples [5,6].

While antimicrobial therapy has significantly reduced TB-related mortality, host-related factors influencing susceptibility and disease progression remain an area of active research. Nutritional status is one such modifiable factor. Undernutrition and poor socioeconomic conditions are established risk factors for TB, largely due to their adverse effects on cell-mediated immunity, a key component of the body’s defense against *M. tuberculosis*. Among nutritional factors, vitamin D has gained considerable attention for its immunomodulatory properties [7,8].

Vitamin D, a steroid hormone primarily involved in maintaining calcium and phosphorus balance, also plays an important role in regulating both innate and adaptive immune responses [9]. The active form of vitamin D enhances macrophage-mediated antimicrobial responses by promoting phagosome-lysosome fusion, thereby limiting intracellular survival of *M. tuberculosis*. It also induces the expression of antimicrobial peptides such as LL-37 (cathelicidin), which form part of the first line of defense against mycobacterial infection. Additionally, vitamin D modulates T-cell activation and antigen presentation, influencing adaptive immune responses. These mechanisms provide biological plausibility for the hypothesis that vitamin D deficiency may increase susceptibility to TB [10].

Vitamin D deficiency is recognized as a widespread public health problem, affecting nearly one billion individuals globally. The major source of vitamin D is its synthesis in the skin following exposure to ultraviolet B radiation, while dietary sources are relatively limited and mainly include fatty fish and fortified foods. Modern lifestyle changes, reduced sun exposure, and environmental factors have contributed to the increasing prevalence of deficiency. In India, the Comprehensive National Nutrition Survey (2016-2018) documented vitamin D deficiency (serum 25(OH)D <12 ng/mL) in 14% of children aged 1-4 years, 18% of school-aged children, and 24% of adolescents, with a higher prevalence among females. Several hospital-based studies have reported even higher rates, especially among children with infectious illnesses [11,12].

Multiple studies have reported that vitamin D deficiency is more common in individuals with pulmonary and extrapulmonary TB than in healthy controls, with prevalence estimates in Asian and African populations ranging from 8.5% to 62.7%. However, most studies have focused on adult populations, and data specifically addressing newly diagnosed childhood TB, particularly in the Indian context, remain limited [13].

Given India’s high TB burden and emerging evidence linking vitamin D to host immune defense against *M. tuberculosis*, evaluating vitamin D status in children with TB is of clinical and public health importance. Understanding this association may help identify children at increased risk and inform adjunct preventive or therapeutic strategies. This study was conducted to determine the prevalence of vitamin D deficiency among children with TB and to evaluate its relationship with demographic and clinical characteristics.

## Materials and methods

Study design and setting

This prospective observational study was conducted in the Departments of Pediatrics, Respiratory Medicine, and Biochemistry at Himalayan Institute of Medical Sciences (HIMS), Dehradun, India. The study was performed over a 12-month period from January 2023 to December 2023 after obtaining ethical approval (SRHU/HIMS/ETHICS/2024/109).

Study population

The study included children aged up to 18 years attending the outpatient and inpatient services of the Departments of Pediatrics, Respiratory Medicine, and Biochemistry at HIMS, Dehradun, and children with recently diagnosed TB, including those receiving anti-tubercular therapy for not more than one month at the time of enrollment.

Sample size

The sample size was estimated using the formula for calculating a single proportion: n = Z² × p × q / d², where n represents the required sample size, Z is the standard normal deviate at a 95% confidence level (1.96), p is the anticipated prevalence of vitamin D deficiency, q = 1 − p, and d is the permissible error. Based on findings from earlier studies, the prevalence of vitamin D deficiency among children with tuberculosis was estimated at 85% [14,15]. Using p = 0.85, q = 0.15, and an absolute precision of 10% (d = 0.10), the estimated sample size was approximately 49 participants. Owing to the time-limited duration of the study, a total of 41 eligible children were ultimately included.

Eligibility criteria

Children up to 18 years of age with newly diagnosed TB or within one month of initiation of anti-tubercular treatment were included in the study. Exclusion criteria included reactivation or recurrence of TB, immunocompromised states, chronic kidney disease, chronic liver disease, parathyroid disorders, chronic lung disease, type I diabetes mellitus, receipt of vitamin D supplementation within the preceding three months, and severe acute malnutrition.

Data collection tools and clinical assessment

Data were collected using a pre-designed and structured questionnaire. The questionnaire captured sociodemographic details assessed according to the Modified Kuppuswamy scale [16], along with clinical information including detailed history, physical examination findings, and relevant laboratory investigations. All enrolled participants underwent a comprehensive clinical evaluation, and any associated comorbid conditions were documented.

Diagnostic evaluation for TB

Diagnostic investigations were recorded for all participants to confirm TB. These included Sputum TRUENAT or Gastric Aspirate TRUENAT, sputum examination for Acid-Fast Bacilli (AFB) or culture, and chest radiography. Additional investigations such as cerebrospinal fluid (CSF) analysis, pleural fluid analysis, lymph node biopsy, or fine-needle aspiration cytology (FNAC) were performed when clinically indicated. Advanced imaging modalities such as chest CT, abdomen CT, or brain CT were carried out where required to support the diagnosis.

Assessment of serum vitamin D levels

Serum vitamin D levels were assessed in all study participants. Venous blood samples were obtained under aseptic conditions and processed by centrifugation prior to analysis. Serum 25-hydroxy vitamin D (25(OH)D) concentrations were determined using the chemiluminescence assay method and expressed in nmol/L (Abbott Architect i2000). Based on serum levels, vitamin D status was classified as deficient (<50 nmol/L) or non-deficient (≥50 nmol/L) [17].

Data management and statistical analysis

Data were recorded in Microsoft Excel 2019 (Microsoft Corp., Redmond, WA, USA) and analyzed using IBM SPSS Statistics for Windows, Version ** (Released Year; IBM Corp., Armonk, NY, USA). Continuous variables were summarized as mean ± standard deviation (SD), whereas categorical variables were expressed as frequencies and percentages. Associations between vitamin D status and categorical variables were evaluated using the chi-square test or Fisher's exact test, as appropriate. Differences in mean serum vitamin D levels between groups were analyzed using the independent t-test and one-way analysis of variance (ANOVA). Pearson correlation analysis was applied to examine the relationship of vitamin D levels with continuous variables such as age and monthly family income. A p-value of less than 0.05 was considered statistically significant.

## Results

Among the 41 study participants, the majority were vitamin D deficient (<50 nmol/L), accounting for 37 (90.2%) individuals with a mean vitamin D level of 31.23 ± 8.47 nmol/L. Only four (9.8%) participants had non-deficient vitamin D levels (≥50 nmol/L), with a higher mean level of 67.40 ± 6.18 nmol/L. The overall mean serum vitamin D level in the study population was 34.75 ± 11.28 nmol/L, indicating a high prevalence of vitamin D deficiency among the participants (Table 1).

**Table 1:** Distribution of vitamin D status among study participants. Data are presented as frequency (n (%)) and mean ± SD.

Vitamin D category	Frequency, n (%)	Vitamin D mean ± SD (nmol/L)
Deficient (<50 nmol/L)	37 (90.2)	31.23 ± 8.47
Non-deficient (≥50 nmol/L)	4 (9.8)	67.40 ± 6.18
Total	41 (100)	34.75 ± 11.28

Vitamin D deficiency was observed across all age groups. The highest proportion of participants belonged to the 11-15 years age group (39.0%), followed by 16-18 years (24.4%), 6-10 years (19.5%), and 0-5 years (17.1%). Among the deficient participants, the largest share was in the 11-15 years group (40.5%). The mean vitamin D level was highest in the 16-18 years age group (38.77 ± 12.19 nmol/L) and lowest in the 11-15 years group (32.73 ± 11.46 nmol/L). However, the association between age group and vitamin D deficiency was not statistically significant (χ² = 2.240, p = 0.524), and there was no significant difference in mean vitamin D levels across age groups (F = 0.598, p = 0.620) (Table 2).

**Table 2:** Association of age group with vitamin D deficiency among study participants. Data are presented as n (%) and mean ± SD. Chi-square test and one-way ANOVA were used. A p-value < 0.05 was considered statistically significant.

Age group	Total, n (%)	Deficient, n (%)	Non-deficient, n (%)	Vitamin D mean ± SD (nmol/L)	Chi-square	ANOVA
0-5 years	7 (17.1)	7 (18.9)	0 (0)	33.63 ± 11.92	χ² = 2.240; p = 0.524	F = 0.598; p = 0.620
6-10 years	8 (19.5)	7 (18.9)	1 (25.0)	34.76 ± 9.99		
11-15 years	16 (39.0)	15 (40.5)	1 (25.0)	32.73 ± 11.46		
16-18 years	10 (24.4)	8 (21.6)	2 (50.0)	38.77 ± 12.19		
Total	41 (100)	37 (100)	4 (100)	34.75 ± 11.28		

Among the 41 participants, females constituted the majority (68.3%) compared to males (31.7%). Vitamin D deficiency was observed in 12 (32.4%) males and 25 (67.6%) females. The mean vitamin D level was slightly higher in males (36.56 ± 8.01 nmol/L) than in females (33.91 ± 12.56 nmol/L). However, there was no statistically significant association between gender and vitamin D deficiency (p = 1.000), and the difference in mean vitamin D levels between males and females was also not statistically significant (t = 0.695, p = 0.491) (Table 3).

**Table 3:** Association of gender with vitamin D deficiency among study participants. Data are presented as n (%) and mean ± SD. Fisher's exact test and independent t-test were used. A p-value < 0.05 was considered statistically significant.

Gender	Total, n (%)	Deficient, n (%)	Non-deficient, n (%)	Vitamin D mean ± SD (nmol/L)	Fisher's exact test	t-test
Male	13 (31.7)	12 (32.4)	1 (25.0)	36.56 ± 8.01	p = 1.000	t = 0.695; p = 0.491
Female	28 (68.3)	25 (67.6)	3 (75.0)	33.91 ± 12.56		
Total	41 (100)	37 (100)	4 (100)	34.75 ± 11.28		

Most participants belonged to the lower middle (34.1%) and lower (31.7%) socioeconomic groups. Vitamin D deficiency was observed across all socioeconomic categories, with the highest proportion among the lower middle group (35.1%), followed by the lower (29.7%) and upper lower (24.3%) groups. The mean vitamin D level was highest in the lower middle group (40.36 ± 7.92 nmol/L) and lowest in the upper middle group (24.89 ± 8.63 nmol/L). However, there was no statistically significant association between socioeconomic status and vitamin D deficiency (χ² = 1.009, p = 0.908), and the difference in mean vitamin D levels across socioeconomic groups was also not statistically significant (F = 1.864, p = 0.138) (Table 4).

**Table 4:** Association of socioeconomic status with vitamin D deficiency among study participants. Data are presented as n (%) and mean ± SD. Chi-square test and one-way ANOVA were used. A p-value < 0.05 was considered statistically significant.

Socioeconomic status	Total, n (%)	Deficient, n (%)	Non-deficient, n (%)	Vitamin D mean ± SD (nmol/L)	Chi-square	ANOVA
Lower	13 (31.7)	11 (29.7)	2 (50.0)	33.02 ± 12.19	χ² = 1.009; p = 0.908	F = 1.864; p = 0.138
Lower middle	14 (34.1)	13 (35.1)	1 (25.0)	40.36 ± 7.92		
Upper lower	10 (24.4)	9 (24.3)	1 (25.0)	30.50 ± 12.05		
Upper	2 (4.9)	2 (5.4)	0 (0)	37.88 ± 15.41		
Upper middle	2 (4.9)	2 (5.4)	0 (0)	24.89 ± 8.63		
Total	41 (100)	37 (100)	4 (100)	34.75 ± 11.28		

Pulmonary TB was the most common diagnosis (41.5%), followed by abdominal TB (29.3%), CNS TB (19.5%), and disseminated TB (9.7%). Among the deficient participants, the highest proportion was observed in pulmonary TB (37.8%), followed by abdominal TB (29.7%), CNS TB (21.6%), and disseminated TB (10.8%). The mean vitamin D level was highest in pulmonary TB (37.52 ± 12.01 nmol/L) and lowest in disseminated TB (24.27 ± 3.74 nmol/L). However, no statistically significant association was found between various types of TB diagnosis category and vitamin D deficiency (χ² = 2.527, p = 0.640), and the difference in mean vitamin D levels across diagnosis categories was also not statistically significant (F = 0.754, p = 0.527) (Table 5).

**Table 5:** Association of tuberculosis (TB) diagnosis category with vitamin D deficiency among study participants. Data are presented as n (%) and mean ± SD. Chi-square test and one-way ANOVA were used for analysis. A p-value < 0.05 was considered statistically significant.

Diagnosis category	Total, n (%)	Deficient, n (%)	Non-deficient, n (%)	Vitamin D mean ± SD (nmol/L)	Chi-square	ANOVA
Pulmonary TB	17 (41.5)	14 (37.8)	3 (75.0)	37.52 ± 12.01	χ² = 2.527; p = 0.640	F = 0.754; p = 0.527
Abdominal TB	12 (29.3)	11 (29.7)	1 (25.0)	32.07 ± 11.54		
CNS TB	8 (19.5)	8 (21.6)	0 (0)	35.08 ± 8.86		
Disseminated TB	4 (9.7)	4 (10.8)	0 (0)	24.27 ± 3.74		
Total	41 (100)	37 (100)	4 (100)	34.75 ± 11.28		

Participants were distributed across different monthly family income categories, with the largest proportion in the ₹7,595-15,196 group (31.7%), followed by ₹5,694-7,594 (22.0%), ₹3,793-5,693 (17.1%), and ≥₹15,197 and ₹2,273-3,792 (14.6% each). Vitamin D deficiency was observed in all income categories, with the highest proportion in the ₹7,595-15,196 group (32.4%). The mean vitamin D level was highest in the ≥₹15,197 income group (36.12 ± 10.54 nmol/L) and lowest in the ₹2,273-3,792 group (32.41 ± 13.26 nmol/L). However, there was no statistically significant association between monthly family income and vitamin D deficiency (χ² = 1.67, p = 0.79), and the difference in mean vitamin D levels across income groups was also not statistically significant (F = 1.173, p = 0.333) (Table 6).

**Table 6:** Association of monthly family income with vitamin D deficiency among study participants. Data are presented as n (%) and mean ± SD. Chi-square test and one-way ANOVA were used for analysis. A p-value < 0.05 was considered statistically significant.

Monthly family income (INR)	Total, n (%)	Deficient, n (%)	Non-deficient, n (%)	Vitamin D mean ± SD (nmol/L)	Chi-square	ANOVA
≥15,197	6 (14.6)	5 (13.5)	1 (25.0)	36.12 ± 10.54	χ² = 1.67; p = 0.79	F = 1.173; p = 0.333
7,595-15,196	13 (31.7)	12 (32.4)	1 (25.0)	34.88 ± 11.74		
5,694-7,594	9 (22.0)	8 (21.6)	1 (25.0)	33.74 ± 9.63		
3,793-5,693	7 (17.1)	6 (16.2)	1 (25.0)	35.09 ± 12.48		
2,273-3,792	6 (14.6)	6 (16.2)	0 (0)	32.41 ± 13.26		
Total	41 (100)	37 (100)	4 (100)	34.75 ± 11.28		

Sputum examination was the most common diagnostic method (39.0%), followed by radiological diagnosis (29.3%) and gastric aspirate (19.5%). Vitamin D deficiency was observed across all diagnostic methods, with the highest proportion among participants diagnosed by sputum examination (37.8%) and radiological methods (29.7%). The mean vitamin D level was highest in microbiology-negative cases (48.78 nmol/L) and lowest in pleural fluid analysis (29.88 ± 6.81 nmol/L). However, there was no statistically significant association between diagnostic method and vitamin D deficiency (χ² = 2.01, p = 0.730), and the difference in mean vitamin D levels across diagnostic methods was also not statistically significant (F = 1.207, p = 0.326) (Table 7).

**Table 7:** Association of diagnostic method with vitamin D deficiency among study participants. Data are presented as n (%) and mean ± SD. Chi-square test and one-way ANOVA were used for analysis. A p-value < 0.05 was considered statistically significant. MRI: magnetic resonance imaging, CECT: contrast-enhanced computed tomography.

Diagnostic method	Total, n (%)	Deficient, n (%)	Non-deficient, n (%)	Vitamin D mean ± SD (nmol/L)	Chi-square	ANOVA
Sputum examination	16 (39.0)	14 (37.8)	2 (50.0)	36.31 ± 10.85	χ² = 2.01; p = 0.730	F = 1.207; p = 0.326
Radiological diagnosis (MRI/CECT)	12 (29.3)	11 (29.7)	1 (25.0)	33.64 ± 11.73		
Gastric aspirate (GA)	8 (19.5)	7 (18.9)	1 (25.0)	34.27 ± 9.44		
Cerebrospinal fluid (CSF) analysis	2 (4.9)	2 (5.4)	0 (0)	32.14 ± 7.63		
Pleural fluid analysis	2 (4.9)	2 (5.4)	0 (0)	29.88 ± 6.81		
Microbiology negative	1 (2.4)	1 (2.7)	0 (0)	48.78		
Total	41 (100)	37 (100)	4 (100)	34.75 ± 11.28		

Pearson correlation analysis showed a weak positive correlation between age and serum vitamin D levels (r = 0.082), which was not statistically significant (p = 0.610). Similarly, monthly family income also showed a weak positive correlation with vitamin D levels (r = 0.088), but this association was not statistically significant (p = 0.584) (Table 8).

**Table 8:** Correlation of age and monthly family income with serum vitamin D levels among study participants. Data are presented as mean ± SD. Pearson correlation analysis was used. A p-value < 0.05 was considered statistically significant.

Variable	Mean ± SD	Correlation (r)	p value
Age	11.44 ± 4.78	0.082	0.610
Monthly income	10036.6 ± 8694.4	0.088	0.584

## Discussion

Vitamin D, an important immunomodulator, has been increasingly studied in relation to TB. However, pediatric evidence remains limited, especially from high-burden countries like India. In the present study, a very high prevalence of vitamin D deficiency (90.2%) was observed among children with TB, with a mean serum 25(OH)D level of 34.75 ± 11.28 nmol/L. Comparable findings have been reported in previous studies, although with some variability in magnitude. Ganmaa et al. reported markedly low vitamin D levels in Mongolian children with TB, with a mean of 17.5 ± 10 nmol/L, which is even lower than that observed in the present study [18]. Similarly, a study among refugee children in Australia found mean vitamin D levels of 35.9 nmol/L in children with TB infection, closely approximating our findings [19].

Other pediatric studies have also demonstrated a high burden of deficiency. Ahmed and Aslam reported that 73.6% of children with TB had low vitamin D levels (deficiency or insufficiency), compared to 47.1% in controls [20]. Rayaz et al. observed 34% severe deficiency and 32% mild deficiency, indicating that nearly two-thirds of pediatric TB patients had suboptimal vitamin D levels [21]. Khandelwal et al. reported that 69.9% of children were vitamin D deficient and 20.7% insufficient, resulting in over 90% having inadequate vitamin D levels [22]. Similarly, Shah et al. documented 85.3% prevalence of vitamin D deficiency or insufficiency among children with TB [14], while Williams et al. reported deficiency or insufficiency in 80% of cases [15]. Parameswaran et al. observed 70% deficiency, 17.5% insufficiency, and only 12.5% sufficiency in children with TB [23]. In contrast, Tha et al. reported a relatively lower prevalence of 49.46% vitamin D deficiency among pediatric TB patients [24].

Adult studies and meta-analyses also support these findings. A systematic review and meta-analysis including 531 participants reported that individuals with low vitamin D levels had a significantly higher risk of active TB [25]. Ho-Pham et al. reported vitamin D insufficiency in 35.4% of men and 45.3% of women with TB [26], which is lower than the prevalence observed in our study, possibly reflecting geographic, nutritional, and methodological differences.

With respect to disease type, previous studies have shown mixed findings. Khandelwal et al. reported vitamin D deficiency in 75% of primary pulmonary complex, 67% of progressive pulmonary disease, and 69% of pleural effusion cases, indicating no major variation across disease types [22]. Likewise, adult studies have shown no significant variation in vitamin D levels between pulmonary and extrapulmonary TB cases. However, Pareek et al. observed lower mean vitamin D concentrations among patients with extrapulmonary TB and reported that a twofold increase in serum vitamin D levels was associated with a lower risk of extrapulmonary disease [27].

The high prevalence of vitamin D deficiency identified in the present study aligns with the overall epidemiological pattern observed in India. Although sunlight is abundant, vitamin D deficiency continues to be common because of factors such as inadequate sun exposure, increased skin pigmentation, poor dietary intake, and limited food fortification practices. The Comprehensive National Nutrition Survey (CNNS) documented vitamin D deficiency in 14% of preschool children, 18% of school-aged children, and 24% of adolescents; however, hospital-based studies, including the current study, have generally reported substantially higher prevalence rates [28].

The variability in prevalence across studies, from approximately 49% to over 90%, may be attributed to differences in study design, cutoff values used to define deficiency, geographic location, nutritional status, and sunlight exposure. Notably, the prevalence observed in the present study (90.2%) lies at the higher end of this spectrum, underscoring the substantial burden in our study population.

In contrast to some earlier studies that reported associations between vitamin D status and disease-related characteristics, the present study did not observe any significant relationship between vitamin D deficiency and the demographic or clinical variables evaluated. No significant associations were identified between vitamin D deficiency and the variables examined; however, the study may have been underpowered to detect modest differences between subgroups.

The study has certain limitations. The study did not achieve the calculated sample size of 49 participants and included only 41 participants. Consequently, the study may have been underpowered to detect modest associations between vitamin D status and demographic or clinical variables. Moreover, important determinants of vitamin D status, including dietary habits, sunlight exposure, seasonal variation, and skin pigmentation, were not evaluated. Detailed anthropometric and nutritional assessments were not available for all participants. Therefore, the potential confounding effect of nutritional status on vitamin D levels could not be evaluated. Because this was a cross-sectional study conducted in children with active TB, reverse causation cannot be excluded. Active illness, inflammation, reduced sunlight exposure, and treatment-related factors may themselves contribute to lower serum vitamin D concentrations. Therefore, the findings should not be interpreted as evidence that vitamin D deficiency causes TB. Direct comparison should be interpreted cautiously because the present study classified vitamin D status using a single threshold of <50 nmol/L, whereas several cited studies reported combined deficiency and insufficiency categories. Differences in classification systems may partially explain variations in reported prevalence. The absence of a healthy control group prevented comparison of vitamin D status between children with TB and the general pediatric population and limited the ability to assess any association between TB and vitamin D deficiency. Further large-scale multicentric studies are warranted to better clarify the role of vitamin D in pediatric TB and to assess the possible benefits of vitamin D supplementation.

## Conclusions

This study revealed a very high prevalence of vitamin D deficiency (90.2%) among children with TB, with a mean serum 25(OH)D level of 34.75 ± 11.28 nmol/L. No significant association was observed between vitamin D deficiency and demographic or clinical factors such as age, sex, socioeconomic status, type of TB, family income, or diagnostic modality. A high prevalence of vitamin D deficiency was observed in this cohort of children with TB. Larger controlled studies are needed to determine the clinical significance of these findings and to evaluate the role of vitamin D assessment and supplementation in pediatric TB.

## References

[1] Chen Y, Chen W, Cheng Z (2024). Global burden of HIV-negative multidrug- and extensively drug-resistant tuberculosis based on Global Burden of Disease Study 2021. Sci One Health.

[2] Al Awaidy S, Khamis F, Al Mujeini S, Al Salman J, Al-Tawfiq JA (2025). Ending tuberculosis in Gulf Cooperation Council countries: an overview of the WHO End TB Strategy 2025 milestones. IJID Reg.

[3] Veggalam S, Kandi V (2026). Elimination of tuberculosis by 2025, an Indian perspective. Discov Public Health.

[4] Jain SK, Ordonez A, Kinikar A (2013). Pediatric tuberculosis in young children in India: a prospective study. Biomed Res Int.

[5] Kashyap B, Gupta N, Dewan P, Hyanki P, Singh NP (2022). Hypovitaminosis D in pediatric tuberculosis: a clinicomicrobiological study. Egypt J Chest Dis Tuberc.

[6] Basu S, Chakraborty S (2025). A comprehensive review of the diagnostics for pediatric tuberculosis based on assay time, ease of operation, and performance. Microorganisms.

[7] Bhargava A, Benedetti A, Oxlade O, Pai M, Menzies D (2014). Undernutrition and the incidence of tuberculosis in India: national and subnational estimates of the population-attributable fraction related to undernutrition. Natl Med J India.

[8] Athanassiou L, Mavragani CP, Koutsilieris M (2022). The immunomodulatory properties of vitamin D. Mediterr J Rheumatol.

[9] Vagha K, Taksande A, Lohiya S, Javvaji CK, Vagha JD, Uke P (2024). Unlocking vitality: a comprehensive review of vitamin D's impact on clinical outcomes in critically ill children. Cureus.

[10] Junaid K, Rehman A (2019). Impact of vitamin D on infectious disease-tuberculosis-a review. Clin Nutr Exp.

[11] Janoušek J, Pilařová V, Macáková K (2022). Vitamin D: sources, physiological role, biokinetics, deficiency, therapeutic use, toxicity, and overview of analytical methods for detection of vitamin D and its metabolites. Crit Rev Clin Lab Sci.

[12] Gupta P, Dabas A, Seth A (2022). Indian Academy of Pediatrics Revised (2021) guidelines on prevention and treatment of vitamin D deficiency and rickets. Indian Pediatr.

[13] Wakayo T, Belachew T, Vatanparast H, Whiting SJ (2015). Vitamin D deficiency and its predictors in a country with thirteen months of sunshine: the case of school children in central Ethiopia. PLoS One.

[14] Shah I, Tolani D, Bansal N, Shetty NS (2019). Vitamin D status in children with tuberculosis. Indian J Pediatr.

[15] Williams B, Williams AJ, Anderson ST (2008). Vitamin D deficiency and insufficiency in children with tuberculosis. Pediatr Infect Dis J.

[16] Saleem SM, Jan SS (2021). Modified Kuppuswamy socioeconomic scale updated for the year 202q1. Indian J Forensic Community Med.

[17] Holick MF, Chen TC (2008). Vitamin D deficiency: a worldwide problem with health consequences. Am J Clin Nutr.

[18] Ganmaa D, Giovannucci E, Bloom BR (2012). Vitamin D, tuberculin skin test conversion, and latent tuberculosis in Mongolian school-age children: a randomized, double-blind, placebo-controlled feasibility trial. Am J Clin Nutr.

[19] Gray K, Wood N, Gunasekera H, Sheikh M, Hazelton B, Barzi F, Isaacs D (2012). Vitamin D and tuberculosis status in refugee children. Pediatr Infect Dis J.

[20] Ahmed M, Aslam M (2015). A study of vitamin D status in children with tuberculosis in Peterborough, United Kingdom. Int J Contemp Pediatr.

[21] Rayaz S, Bizenjo A, Hussain M, Iqbal M (2022). Frequency of vitamin D deficiency in children with tuberculosis. Pak J Med Health Sci.

[22] Khandelwal D, Gupta N, Mukherjee A (2014). Vitamin D levels in Indian children with intrathoracic tuberculosis. Indian J Med Res.

[23] Parameswaran P, Vaidya PC, Attri SV, Angurana SK, Vignesh P, Singh M (2021). Vitamin D deficiency: prevalence and association with intrathoracic tuberculosis in Indian children. Indian J Pediatr.

[24] Tha S, Rasheed S, Mukhtar A, Rasheed M, Naz S, Javaid MF (2022). Vitamin D deficiency in children with tuberculosis: a cross sectional study. Pak J Med Health Sci.

[25] Nnoaham KE, Clarke A (2008). Low serum vitamin D levels and tuberculosis: a systematic review and meta-analysis. Int J Epidemiol.

[26] Ho-Pham LT, Nguyen ND, Nguyen TT, Nguyen DH, Bui PK, Nguyen VN, Nguyen TV (2010). Association between vitamin D insufficiency and tuberculosis in a Vietnamese population. BMC Infect Dis.

[27] Pareek M, Innes J, Sridhar S (2015). Vitamin D deficiency and TB disease phenotype. Thorax.

[28] Rana G, Abraham RA, Sachdev HS (2023). Prevalence and correlates of vitamin D deficiency among children and adolescents from a nationally representative survey in India. Indian Pediatr.

